# A quadrigeminal arachnoid cyst as a cause of neurological symptoms in an 11-month-old Brussels Griffon – A case study

**DOI:** 10.17221/53/2023-VETMED

**Published:** 2023-08-31

**Authors:** Marta Mieszkowska, Korina Michalska, Marcin Mieszkowski, Michalina Dowgierd, Yauheni Zhalniarovich

**Affiliations:** ^1^Department of Surgery and Radiology with Clinic, Faculty of Veterinary Medicine, University of Warmia and Mazury in Olsztyn, Olsztyn, Poland; ^2^Department of Anesthesiology and Intensive Care, Faculty of Medicine, Collegium Medicum, University of Warmia and Mazury in Olsztyn, Olsztyn, Poland; ^3^Student of the 4^th^ year, Faculty of Veterinary Medicine, University of Warmia and Mazury in Olsztyn, Olsztyn, Poland

**Keywords:** intracranial cyst, congenital brain malformation, MRI

## Abstract

An intracranial arachnoid cyst (IAC) is a rare developmental disorder that is consistent with cerebrospinal fluid accumulation between the brain and the arachnoid membrane. A quadrigeminal cyst is a specific type of cyst that is identified based on its localization. To the best of our knowledge, this is the first study to report on this type of pathology in a Brussels Griffon. This case study describes an 11-month-old female Brussels Griffon with symptoms of reluctance to lower the head and eat from a bowl placed on the ground, combined with episodes of vocalization. The patient was subsequently diagnosed with a quadrigeminal cyst during a low-field magnetic resonance imaging (MRI) exam, and she tested positive for toxoplasmosis in the blood test. Arachnoid cysts are often described as incidental findings, but the characteristics of neurological symptoms in the presented patient suggest that the cyst was clinically significant. The currently known options of pharmacological and surgical treatment give some hope for symptomatic patients, although their definitive success rate is not yet fully known.

Arachnoid cysts are developmental anomalies that are often diagnosed as incidental findings with no clinical signs (Matiasek et al. 2007). Their congenital nature is attributed to the ectopic choroid plexus or ependymal cells which cause the secretion of cerebrospinal fluid (Osborn 1994) and arise from the splitting or duplication of the arachnoid in the embryonic period (Renganchary and Watanabe 1981). Some arachnoid cysts might have a traumatic origin, and they haemorrhage to the subarachnoid space (Renganchary and Watanabe 1981). Typically, arachnoid cysts lack epithelial lining (Dyce et al. 1991), and the term “cyst” is misleading, but generally accepted (Skeen et al. 2003). A quadrigeminal cistern is a dilatation of the subarachnoid space between the corpus callosum and the cerebellum. It is localized between the layers of the *tela choroidea* of the third ventricle. A quadrigeminal arachnoid cyst is a cyst with a pathognomonic infratentorial location between the cerebral occipital lobe and the rostral part of the cerebellum (Dewey et al. 2009). Cysts are most often found in canine patients of small and brachycephalic breeds (Vernau et al. 1997). Most cases of subarachnoid cysts were described in Shih Tzu dogs (Matiasek et al. 2007), followed by Maltese, Pugs, Lhasa Apso, Cavalier King Charles Spaniels, and Yorkshire Terriers. In both humans and animals, a sexual predilection has been noted, with an increased incidence of subarachnoid cysts among male individuals (Pascual-Castroviejo et al. 1991). In humans, the prevalence of cysts was determined at 1.8% in males and 1.1% in females (Al-Holou et al. 2013), and it was also found to be higher in male dogs (Matiasek et al. 2007). This clinical case study describes an 11-month-old female dog that was diagnosed with a quadrigeminal cyst during a magnetic resonance imaging (MRI) exam, and to the best of our knowledge, this is the first published report of a quadrigeminal cyst in a Brussels Griffon.

## Case presentation

An 11-month-old female dog was admitted to the primary care clinic after the owners had observed a peculiar, stiff upward lifting of her head and paroxysmal vocalization for two days. The dog was reluctant to eat from a bowl placed on the ground, while her appetite was preserved, and she readily ate from a bowl placed at the level of her head. The patient’s consciousness was preserved during vocalization episodes. She defecated and urinated properly and showed no signs of limb paralysis. The results of a neurological exam are summarised in Table 1.

**Table 1:** The results of a neurological exam of an 11-month-old Brussels Griffon

Consciousness	Preserved, patient active
Cranial nerves	Pupils of equal shape and size Pupillary reflexes bilaterally normal Eyelid gaps of the same size Eyeballs in physiological position, right-sided vertical strabismus after lifting the head upwards Eyelid reflexes, corneal reflexes, and threatening reflexes bilaterally normal Normal formation and tension of the mandibular muscles Physiological resistance when opening the mouth Normal sensation in the head area Normal response to irritation of the nasal vestibule
Senses	Vision bilaterally preserved Normal response to acoustic stimuli
Body posture	Physiological in motion and at rest
Skeletal muscles	Normally developed Symmetrical Non-painful Physiological quadriceps tension
Posture and stance reactions	Normal corrective response in four limbs Normal bouncing response in four limbs
Spinal reflexes	Normal flexor muscles and reflex in all extremities Quadriceps thigh muscle reflex bilaterally normal Tibialis occipitalis muscle reflex bilaterally normal Radial extensor muscle wrist reflex bilaterally normal Normal musculocutaneous reflex Normal perineal reflex Physiological anal sphincter muscle tension
Assessment for pain	Full range of passive neck motion preserved Absence of soreness during palpation of the thoracolumbar spine Superficial pain sensation preserved in quadriplegia

After the first neurological exam, the owners were advised to administer pyralginum syrup (500 mg/ml – 0.2 ml, 2× daily) for 3 days (or longer if the pain persisted), CBD oil (10%, 2 drops 2× daily), and gabapentin (100 mg, ¼ tablet 3× daily) to the patient. The blood test revealed elevated leukocyte (14.00 × 10^9^/l; normal range: 6.00–12.00 × 10^9^/l), lymphocyte (56%; normal range: 12–30%), ALT (2.07 μkat/l; normal range: 0.08–1.0 μkat/l), AP (2 850 μkat/l; normal range 83.34–2 583 μkat/l), and calcium (2.9 mmol/l; normal range: 2.10–2.88 mmol/l) levels. Five days later, during a follow-up neurological exam, the patient presented with neurological deficits in postural responses and mild hypermetria. Due to nonspecific symptoms and elevated inflammatory parameters, blood samples were collected for a toxoplasmosis test which returned a positive result. The patient was referred for an MRI scan to evaluate the brain for developmental pathologies and/or pathologies associated with toxoplasmosis.

## MAGNETIC RESONANCE IMAGING (MRI)

Magnetic resonance imaging of the brain was performed in an Esaote Vet Grande (Italy) MRI machine (0.25 T). T1- and T2-weighted images were acquired with the use of the following protocols: SE T1 sag, SE T1 tra, FSE T2 sag, FSE T2 tra, FSE T2 dor, FLAIR dor, and post-contrast sequences after intravenous injection of gadoteric acid at 0.1 mmol/kg (Clariscan, 0.5 mmol/ml): SE T1 tra, SE T1 dor.

**Figure 1:**
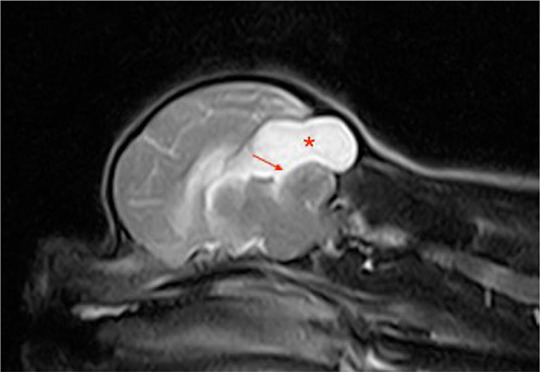
T2 FSE sagittal view with a visible hyperintense cyst (asterisk) compressing the cerebellum cranially (arrow)

Based on the obtained images, a clearly demarcated extra-axial fluid reservoir-like lesion was identified between the occipital lobe of the cerebrum and the cerebellum with prominent cranial compression of the cerebellum (Figure 1).

**Figure 2:**
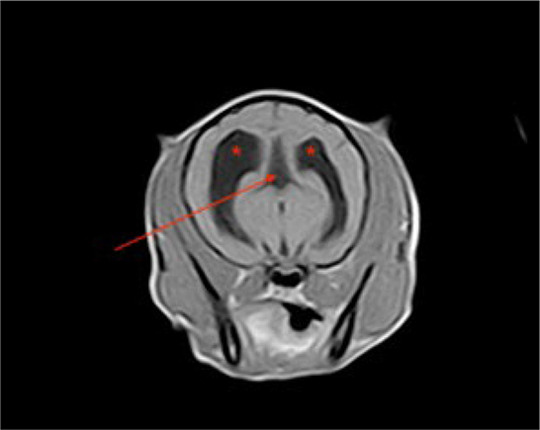
T1 SE transverse view – Dilatation and moderate asymmetry (asterisk) of the lateral ventricles. The triangular hypointense structure between the lateral ventricles and the occipital lobes is consistent with a quadrigeminal cyst (arrow)

The lesion measured 3.7 cm CrCd (craniocaudal) × 1.7 cm RL (right–left) × 2 cm DV (dorsoventral). In addition, there was asymmetry of the lateral ventricles and significant ventricular dilatation with a VH/BH ratio of 73% for the right ventricle and 64% for the left ventricle (Figure 2).

**Figure 3:**
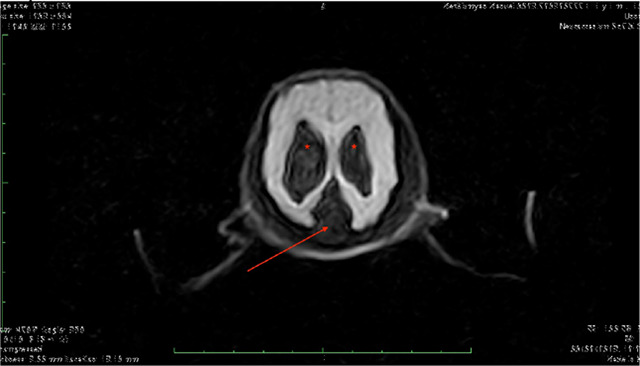
FLAIR dorsal sequence with hypointense fluid within dilatated lateral ventricles (asterisk) and a triangular quadrigeminal cyst localized between the occipital lobes (arrow)

The height of the corpus callosum was 8 mm. The height of the interventricular fusion was 4.3 mm. The third ventricle was dilatated. Moderate dilatation of the fourth ventricle and moderate dilatation of the cerebellar cistern were observed. No features of cerebellar herniation into the foramen magnum were visible. No multifocal hyperintense lesions were found in the cerebellum. There was no pathological signal enhancement in the cerebellar area after the intravenous administration of the paramagnetic contrast agent. The MR image was consistent with significant ventriculomegaly and the presence of a cyst in the quadrigeminal cistern (Figure 3).

## DISCUSSION

In humans, the incidence of subarachnoid cysts is estimated at 1% (Robinson 1971). In dogs, few cases of subarachnoid cysts have been described, and their prevalence has been established at 0.7% (Matiasek et al. 2007).

Differential diagnoses of quadrigeminal cisterna arachnoid cysts include the Dandy-Walker malformation, mega cisterna magna, retrocerebellar arachnoid cyst, and retrocerebellar arachnoid pouch. In humans, the Dandy-Walker malformation is a dilatation of the fourth ventricle and a displacement of the cerebellum with vermian agenesis and the enlargement of the posterior fossa (Friede 1989). In the presented case, the fourth ventricle was only mildly enlarged, and the cerebellum was compressed rather than displaced due to agenesis. The cisterna magna is a cystic malformation combined with the enlargement of both the cisterna magna and the posterior fossa with an intact cerebellum that can be compressed due to the size of the cyst (Raybaud 1982).

However, in the discussed case, the cyst was localized differently than a quadrigeminal cyst, and it spread caudally and ventrally to the cerebellum. A retrocerebellar arachnoid cyst is a malformation that occurs during embryonic development due to the splitting or duplication of the arachnoid membrane which becomes filled with fluid and develops a cystic appearance (Cincu et al. 2007). The analysed cyst was localized differently. A retrocerebellar cyst is localized directly caudally to the cerebellum, and it may compress the cerebellum caudally when it increases in size (Lee et al. 2020).

Most arachnoid cysts remain asymptomatic and stable in size, but some may become symptomatic when accompanied by hydrocephalus or when the cyst becomes enlarged and compresses specific brain structures (Al-Holou et al. 2013; Melicher et al. 2023). In a case study of human subjects, a hydrocephalus-associated quadrigeminal cyst was caused by aqueductal stenosis (Nishida et al. 1988).

The described patient developed cerebellar symptoms shortly before being referred for an MRI scan. The symptoms were initially linked with the toxoplasmosis infection diagnosed in the blood test. In dogs, toxoplasmosis is rarely a primary infection, and the clinical presentation is usually caused by immunosuppression and the absence of vaccination against the canine distemper virus (CDV) (Calero-Bernal and Gennari 2019). The symptoms include seizures, tremors, ataxia, paresis, and paralysis (Patitucci et al. 1997). In the described case, the symptoms of ataxia and hypermetria may have been related to the pressure exerted by an enlarged cyst on the cerebellum. The MRI scan revealed no inflammatory features that could be associated with toxoplasmosis (Coelho et al. 2019; Awang Senik et al. 2023).

Definitive confirmation of toxoplasmosis can be a diagnostic challenge, especially since the MR image of infection is nonspecific, and the radiological signs mentioned in the literature, such as multifocal intracranial hyperintense areas in the cerebellum, periventricular white matter, midbrain, and diencephalon, are not definitively confirmed histopathologically (Lamb et al. 2005). None of these radiological signs were found in the described case, which suggested that the clinical symptoms were directly linked with the presence of a large cyst compressing the cerebellum. The detection of a *Toxoplasma gondii* infection in cerebrospinal fluid in the PCR test would be crucial for the final diagnosis and treatment.

However, due to the presence of a congenital malformation, the owners refused to subject the dog to further diagnosis and treatment. In view of the clinical picture and the results of brain imaging, it can be assumed that the symptoms were largely caused by hydrocephalus and the presence of cysts. According to Matiasek et al. (2007), clinical signs appear in dogs with cerebellar compression of >14%.

In the presented case, the degree of compression was 29%, which may further confirm the clinical significance of intracranial arachnoid cyst (IAC). Concomitant hydrocephalus could be an additional factor responsible for the clinical signs. Hydrocephalus can be a congenital malformation in toy breed dogs (Przyborowska et al. 2013), but in IAC patients, it can be caused by cerebral compression and disrupted flow of the cerebrospinal fluid (Raffel and McComb 1988). Alves et al. (2018) reported hydrocephalus in 20 out of 26 dogs (77%) that were simultaneously diagnosed with IAC.

A similar radiological sign was found in the reported case. Typical signs of hydrocephalus include altered mental states, loss of consciousness, circling, incoordination, seizures, blindness, strabismus, and dilated pupils. In the presented case, none of those symptoms were observed in the neurological exam, but the owners described episodes of paroxysmal vocalization which they associated with presumed pain. Upon the neurological exam, the patient was pain-free and did not present with any of the symptoms that could be linked with hydrocephalus. Therefore, in the authors’ opinion, the symptoms associated with IAC are clinically dominant.

In conclusion, it could be stated, that a quadrigeminal arachnoid cyst is a rare congenital malformation that may remain asymptomatic until it becomes enlarged and contributes to the presentation of clinical symptoms.

The presence of antibodies against *Toxoplasma gondii* in the described case does not indicate an active phase of the disease, but it could result from contact with the pathogen. A cerebrospinal fluid analysis would be necessary to confirm the infection, but it was not performed on the patient due to the lack of owner consent.

## Conflict of interest

The authors declare no conflict of interest.
